# Apheresis in Autoimmune Encephalitis and Autoimmune Dementia

**DOI:** 10.3390/jcm9092683

**Published:** 2020-08-19

**Authors:** Rosa Rössling, Harald Prüss

**Affiliations:** 1 Department of Neurology and Experimental Neurology, Charité–Universitätsmedizin Berlin, Charitéplatz 1, 10117 Berlin, Germany; rosa.roessling@charite.de; 2 German Center for Neurodegenerative Diseases (DZNE) Berlin, 10117 Berlin, Germany

**Keywords:** autoimmune encephalitis, limbic encephalitis, NMDAR (N-Methyl-D-Aspartat), antibody, paraneoplastic, apheresis, plasma exchange, immunoadsorption

## Abstract

Autoimmune encephalitis (AE) is a rapidly progressive inflammatory neurological disease. Underlying autoantibodies can bind to neuronal surfaces and synaptic proteins resulting in psychiatric symptoms, focal neurological signs, autonomic dysfunction and cognitive decline. Early and effective treatment is mandatory to reduce clinical symptoms and to achieve remission. Therapeutic apheresis, involving both plasma exchange (PE) and immunoadsorption (IA), can rapidly remove pathogenic antibodies from the circulation, thus representing an important first-line treatment in AE patients. We here review the most relevant studies regarding therapeutic apheresis in AE, summarizing the outcome for patients and the expanding clinical spectrum of treatment-responsive clinical conditions. For example, patients with slowly progressing cognitive impairment suggesting a neurodegenerative dementia can have underlying autoantibodies and improve with therapeutic apheresis. Findings are encouraging and have led to the first ongoing clinical studies assessing the therapeutic effect of IA in patients with anti-neuronal autoantibodies and the clinical presentation of dementia. Therapeutic apheresis is an established and well tolerated option for first-line therapy in AE and, potentially, other antibody-mediated central nervous system diseases.

## 1. Introduction

Autoimmune encephalitis (AE) is a rapidly progressive inflammatory neurological disease with subacute onset. Patients may present with behavioral changes and altered mental status as well as reduced levels of consciousness and new focal neurological signs or epileptic seizures [1]. Furthermore, deficits in working or short-term memory frequently occur.

AE comprises both, antibody-mediated and paraneoplastic, i.e., cytotoxic T-cell-mediated, encephalitides. Clinical presentation is diverse and depends on the specific underlying antibody (Table 1). As more and more novel antibodies and new clinical phenotypes are being identified, the incidence is rising and currently estimated at 5–10 per 100,000 inhabitants per year [1]. Age and gender preferences are often specific for a given antibody. In some cases, the exact target of novel antibodies is not known yet. In other cases, even if the underlying antigen is known, the pathogenic relevance still awaits scientific clarification.

### 1.1. Antibody-Mediated AE

The most common and best-known form of antibody-mediated AE is NMDA (N-Methyl-D-Aspartat) receptor (NMDAR) encephalitis, defined by cerebrospinal fluid (CSF) IgG antibodies targeting the NMDA type glutamate receptor. Patients present with subacute onset of psychiatric symptoms, autonomic instability, focal neurological signs and behavioral changes as well as new-onset epileptic seizures and reduced levels of consciousness. Other AE-defining autoantibodies bind directly to excitatory transmitter receptors besides NMDAR (such as AMPA (**α**-amino-3-hydroxy-5-methyl-4-isoxazolepropionic acid) receptors), inhibitory transmitter receptors (GABAB (gamma-aminobutyric acid B), GABAA (gamma-aminobutyric acid A), glycine receptors), ion channel subunits and cell adhesion molecules (Caspr2 (contactin-associated protein 2), IgLON5) or soluble synaptic proteins (LGI1 (leucine-rich, glioma inactivated protein 1).

Autoimmune dementia might be considered a sub-form of AE with predominant cognitive deficits. Cognitive impairment is a common feature in AE. For instance, patients with encephalitis caused by LGI1 antibodies showed markedly impaired verbal and visuo-spatial memory as well as a significantly reduced hippocampal volume. A severe clinical course correlated with more pronounced structural damage of the hippocampus and correspondingly a worse overall memory performance [2]. As patients show good response to immunotherapy, especially in the early stage of disease, prompt and sufficiently “aggressive” treatment including apheresis is highly important. Interestingly, the cognitive deficits in LGI1 encephalitis can come in isolation and lead to the working diagnosis of a primary neurodegenerative disease such as Alzheimer’s. Increasing awareness and the search for autoantibodies such as LGI1 are needed and can result in the early identification of dementia patients with an immunotherapy-responsive phenotype [3,4].

### 1.2. Paraneoplastic AE

In contrast to the neuronal surface antibodies, antibodies in classical paraneoplastic neurological syndromes (PNS) bind to intracellular antigens (such as Hu, Ri, Yo or Ma2 antibodies) and therefore do not cause the neurotoxicity directly; they rather serve as valuable biomarkers for an underlying tumor, often small cell lung cancer and gynecological tumors. The neuronal damage in these cases is, rather, caused by cytotoxic T-cells with oligoclonal T-cell receptor expansion and autoreactivity against neuronal structures. Among the antibodies targeting intracellular antigens, GAD (glutamic acid decarboxylase) and amphiphysin antibodies are an exception as they are not necessarily associated with a tumor and seem to be pathogenically relevant despite their intracellular antigen location [5].

### 1.3. Therapy for AE

At this point, there is no clear evidence-based treatment standard for AE. Established treatment strategies for first-line therapy of AE include high-dose corticosteroids (three to five days course of 1000 mg intravenous methylprednisolone), intravenous immunoglobulins (IVIG) (2 g/kg body weight over three to five days), as well as therapeutic apheresis. Cyclophosphamide and the CD20-antibody rituximab (1000 mg, with the first two administrations at day 1 and day 15 followed by six months intervals) might be added in case of persisting or relapsing symptoms and as a long term maintenance therapy. Most centers favor a low threshold for rituximab initiation given its good safety profile and potential effect in preventing relapses. Many other treatments have been used with variable success, including mycophenolate mofetil, methotrexate or azathioprine. It is broadly agreed that immunotherapy needs to be started as early as possible after symptom onset to be most effective. Nevertheless, marked recovery can be seen in some patients with antibody-mediated AE in whom therapy is only started months after disease onset. The choice of adequate therapy depends on the clinical syndrome and the underlying antibody. However, comparative treatment studies in patients with AE are sparse and focus on the most common forms of AE, such as NMDAR encephalitis.

In paraneoplastic AE with antibodies targeting intracellular proteins, rituximab, intravenous immunoglobulins and therapeutic apheresis often have only little effect as the antibodies are not directly pathogenic. Here, neuronal damage is caused by cytotoxic T-cells. Evidence of a tumor requires prompt and complete removal in order to withdraw the auto-antigen expressed by the tumor that triggers the production of autoantibodies. Nevertheless, despite advanced immunotherapy and tumor removal, in many cases neuronal damage in paraneoplastic AE progresses.

Therapeutic apheresis and the removal of autoantibodies is a major therapeutic option in AE. The pathophysiological binding of antibodies to their antigens can thereby be reduced.

## 2. Search Strategy

To conduct the review, we followed the PRISMA (Preferred Reporting Items for Systematic Reviews and Meta-Analyses) guidelines and screened the articles independently for their respective eligibility [12].

### 2.1. Inclusion Criteria

We included all articles about patients with autoimmune encephalitis—antibody-mediated as well as paraneoplastic—treated with plasma exchange or immunoadsorption. Treatment regimen, such as concomitant immunotherapy, as well as details about the apheresis itself (plasma exchange (PE) or immunoadsorption (IA), number of courses) had to be specified in the article. Further, outcome measures, such as the modified Rankin Scale (mRS) or structured neuropsychological assessment had to be provided. The mRS ranges from zero (no symptoms) to six (death from the disease), and a change of ±1 mRS point is considered as clinically significant improvement or deterioration. Cut-off for independent living is at ≤2 mRS points.

### 2.2. Search Strategy

**Figure 1:**
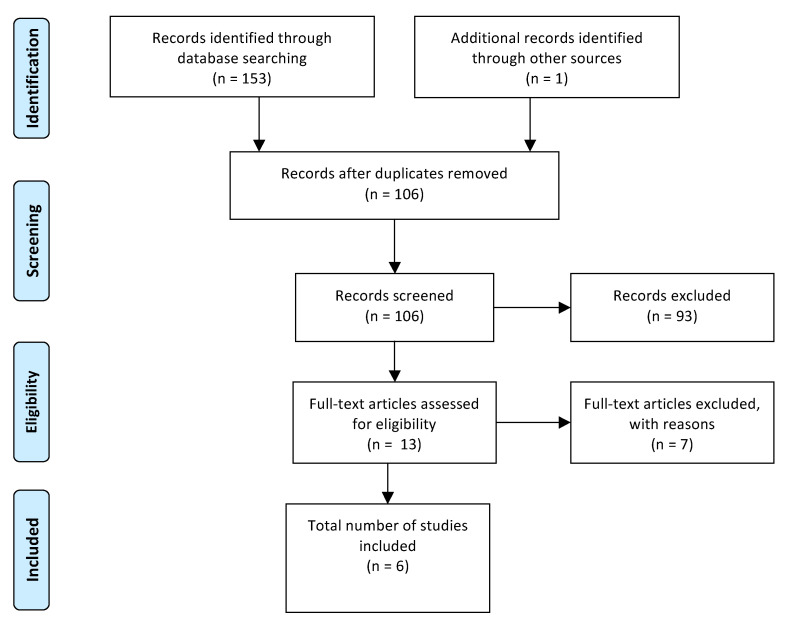
Preferred Reporting Items for Systematic Reviews and Meta-Analyses (PRISMA) flow diagram of the reviewed literature.

The following strategy was used to find previous literature and trials (Figure 1): MEDLINE (medical literature analysis and retrieval system online) was searched for articles published up until 30 June 2020 in English or German. The Medical Subject Headings (MeSH) terms used were “autoimmune encephalitis” and “apheresis” (37 hits), “plasma exchange” (104 hits) or “immunoadsorption” (12 hits). Furthermore, the references of the included articles were screened for potential additional articles.

## 3. Results

### 3.1. Therapeutic Apheresis in Autoimmune Encephalitides

Therapeutic apheresis is an important treatment option in a range of inflammatory central nervous system diseases [13]. It has been proven to be beneficial in primary demyelinating disease as well as in encephalitis caused by antibodies targeting neuronal proteins [14] (Table 2).

Therapeutic apheresis has been shown to be safe and effective leading to measurable laboratory and clinical improvement in several inflammatory diseases of the central and peripheral nervous system, including myasthenia gravis, Guillain-Barré syndrome and multiple sclerosis. Apheresis is recommended by the German Society of Neurology as escalation treatment of severe courses of AE. Patients should be treated with apheresis at least five times every other day. In cases with predominant CSF antibodies seven to ten treatment courses are usually needed for relevant reduction of CSF antibody titers. Before receiving therapeutic apheresis, patients mostly show either severe clinical symptoms on hospital admission or an insufficient response to therapy with high-dose cortisone or IVIG.

Much has been learned from acquired myasthenia gravis, which represents a “model disease” for the much later discovered forms of autoantibody-mediated AE. It could first be demonstrated that removal of the disease-defining acetylcholine receptor antibodies using plasma-exchange led to marked symptom improvement [15]. Antibodies in antibody-mediated AE are mostly directed against neuronal surface antigens. Emerging studies have demonstrated that clinical symptoms relate directly to pathogenic autoantibodies. For example, isolated human monoclonal autoantibodies from patients with NMDAR encephalitis targeted the NR1 subunit of the NMDAR and were alone sufficient to induce morphological and electrophysiological changes in the neurons, and to lead to synaptic dysfunction by downregulation of NMDAR [16]. Thus, the pathogenic effect is caused by the antibodies themselves, indicating that removal of these antibodies can disrupt the disease-causing mechanisms.

Therapeutic apheresis has been shown to improve clinical symptoms in different antibody-mediated diseases. According to the American Society for apheresis (ASFA) guidelines PE and IA are strongly recommended for different antibody-mediated encephalitis forms ranging from low to moderate evidence. In contrast, the therapeutic role of apheresis is not yet established for paraneoplastic neurological syndromes and individual decision-making is necessary [23]. In NMDAR encephalitis, recovery and symptom remission often correlate with a reduction of antibodies, in particular with a decline in CSF titers. In this way, antibody titers can serve as intra-individual disease biomarkers and support treatment decisions [24]. Efficacy of therapeutic apheresis relates to the extracorporeal elimination of circulating serum antibodies, redistribution of antibodies from the extracellular space and a number of secondary immunomodulatory changes. The inflammatory processes during AE are likely to involve a leakier blood-brain barrier, which might support further redistribution of autoantibodies from the central nervous system into the blood [25].

### 3.2. Therapeutic Procedure for Apheresis

Therapeutic apheresis offers two different procedures. On the one hand is plasma exchange (PE), where a defined plasma volume is removed and replaced by human albumin or fresh frozen plasma. On the other hand is immunoadsorption (IA), a procedure that more specifically removes immunoglobulins and immune complexes by passing the plasma over an adsorber column, allowing reinfusion of the patients’ own plasma. Two different IA procedures were used in the reviewed articles: either a regenerative double column system or a disposable tryptophan column. Tolerability and therapeutic effects do not show relevant differences between PE and IA in recent studies [14,19,26]. Related to the procedure is a rare risk of pathogen transmission in PE due to substitution with donor-derived blood components, which is not existent in IA [19]. However, angiotensin-converting enzyme inhibitors need to be paused for a minimum of 48 h prior to IA, otherwise there exists a risk of IA-associated bradykinin-release syndrome. Main side effects are not caused by the apheresis directly, but are rather related to the necessary central venous catheter. They include bleedings, hematoma, infections, thrombosis or damage caused by the puncture [22].

Usually a minimum of five sessions of apheresis is performed. When patients show a CSF predominant antibody, more sessions are generally needed in order to eliminate the antibody in the central nervous system. Most studies included in this review describe a central venous catheter in an internal jugular vein as vascular access. Only Hempel et al. use a peripheral vein to perform IA and in order to treat patients as outpatients. However, they report significant patient drop-out due to a failure of repeatedly accessing the vein [20].

The treated plasma volumes can be calculated using Sprenger’s formula [27]. Depending on the protocol, a total of 1.5–2.2 plasma volume is processed in PE, whereas in IA 2000–2500 mL plasma per session are treated [19,22]. Patients treated with PE receive a replacement solution, such as 4% human albumin or fresh frozen plasma. Treatments take place every other day, although the first two to three sessions can be conducted on consecutive days in selected cases. Due to the central venous catheter, anticoagulation is necessary to minimize the risk of thrombosis.

### 3.3. Initiation of Therapy with Apheresis and Prior Treatment

The specific mechanism of antibody removal has been shown to be a more beneficial treatment of NMDAR encephalitis than intravenous methylprednisolone alone. In a retrospective study 2/14 patients showed significant clinical improvement following steroids, whereas 9/14 patients who received additional PE improved in the mRS during the third and fifth cycle of apheresis [17]. This is likely to be related to the high therapeutic specificity and therefore efficacy of therapeutic apheresis compared to intravenous immunoglobulins (IVIG) or high-dose corticosteroids. Early diagnosis and prompt start of a sufficiently “aggressive” therapy are mandatory for symptom reduction and long-term remission. Interestingly, a study by Heine et al. showed that treatment delay was not associated with a significantly worsened outcome [19], whereas Onugoren et al. found that, in patients with irreversible damage of brain structures, such as fixed hippocampal sclerosis, no clinical improvement could be achieved by IA [22].

Many patients with AE treated with apheresis receive prior treatment with high-dose steroids or IVIG. The decision for treatment with apheresis is often only made after unsuccessful or incomplete recovery after these other therapies. It has been shown that both patients who did and patients who did not receive prior treatment benefitted from apheresis [19]. In all studies analysed, a substantial part of the patients (up to more than half) received apheresis as initial treatment.

In the study by Onugoren et al. all patients except one out of 19 were treated with high-dose prednisolone (median dose 4.9 g) in parallel to IA [22]. It is reported that in some patients immunosuppressive therapy with steroids is continued on a maintenance dose [18]. Especially, patients with antibodies to LGI1 respond well to continued treatment with steroids [8,9].

Apheresis in patients with AE is most established in the acute phase of the disease. However, repeated apheresis might also be applied in refractory disease with clinical signs of AE and constant detection of high antibody titers. Yet in one study, repeated IA after 4.5 months (median) in six patients with antibodies against NMDAR, LGI1, Caspr2 and GAD that responded insufficiently to a first series of IA, did not show any further clinical improvement measured by mRS [22].

In case of an underlying malignancy, tumor removal is essential for improving further disease course.

### 3.4. Effects of Treatment with Apheresis in Patients with AE

A better outcome in patients with NMDAR encephalitis was strongly associated with an early start of immunotherapy (less than 40 days after symptom onset) [28]. Response rate in general is considerably higher when therapy initiation is started early [29] and includes improvement in state-of-the-art imaging and neuropsychological assessments [30].

According to a prospective study, symptoms that responded best to apheresis include apathy, aphasia, stupor, sleep disorders, agitation, myoclonus and dystonia, sensory neuropathy, apraxia and seizures [19]. In another study, in the majority of patients the modified Rankin Scale (mRS) improved by ≥1 point. It is of note that no patient worsened during apheresis in these studies. 

Treatment efficacy is more pronounced in patients with antibodies against cell surface antibodies (NMDAR, LGI1, Caspr2, mGluR5) or in patients with intracellular synaptic antibodies (GAD), whereas no positive treatment effect was observed in patients with paraneoplastic intracellular antigens (anti-Hu) [19,22]. Marked reduction of serum antibodies occurred during the first five sessions of IA, but titers dropped further when apheresis was continued. Five days after IA a median decrease in titers of 97% and 64% was noted for serum and CSF, respectively. Interestingly, the decrease further continued until the next follow-up (median time after IA 3.9 months) [22]. In a retrospective study, all cerebral magnetic resonance imaging (MRI) changes in 17 patients with NMDAR encephalitis decreased [18].

Marked and rapid effects of apheresis can be seen in patients with epileptic seizures concerning seizure frequency. This was seen for patients with LGI1 and Caspr2 antibodies, where five out of seven patients became seizure free immediately after initiation of therapy with IA [22]. This treatment effect results in reduction or even complete removal of antiepileptic drugs.

Several case reports point to the efficacy of PE in drug-resistant status epilepticus caused by AE in children and adults. Both convulsive and non-convulsive status have been described as being responsive to apheresis. In patients with abnormal electroencephalogram (EEG) prior to apheresis, EEG normalization was observed after 5 cycles of PE. EEG improvement correlated with decrease in antibody titers [31,32].

Treatment of severe AE complicated by status epilepticus or autonomic instability might need to take place on an intensive care unit. On the ICU, benefit from immunotherapy, including apheresis, strongly depends on medical complications associated with a prolonged ICU stay [33].

In patients with predominantly psychiatric symptoms related to treatable autoimmunity, corticosteroids are often hesitantly used given the potential side effect of steroid-induced psychosis. Overall, notable neuropsychiatric side effects can occur in up to 6% of patients who receive steroids [34]; however, in antibody-mediated AE the clinical improvement with immunotherapy quickly outrivals any steroid-related effects on psychiatric symptoms according to our experience.

In paraneoplastic AE with antibodies targeting intracellular proteins, therapeutic effects of rituximab, IVIG and therapeutic apheresis are usually limited as the antibodies are not directly pathogenic, but neuronal damage is caused by cytotoxic T-cells [19,26]. Furthermore, diagnosis in paraneoplastic disease is often delayed and substantial irreversible neuronal cell damage has already occurred at the time of therapy initiation. Discontinuation of therapy should be considered in patients in whom brain damage has progressed to an advanced stage in MRI after three to six months despite intensified immunotherapy.

After therapeutic apheresis with both PE and IA, a transient spurious intrathecal immunoglobulin synthesis of all three subclasses (IgG, IgA, IgM) can be observed. The transient intrathecal Ig fractions and increased IgG index are due to dropped serum IgG levels following apheresis. This “intrathecal pseudo-synthesis” regularly occurs in the first two days after apheresis in a majority of patients [35]. Thus, one needs to consider these abnormalities for interpretation of CSF results from lumbar puncture shortly after apheresis in order to prevent false diagnostic assumptions.

### 3.5. Future Treatment Options for Apheresis

Clinical indications for therapeutic apheresis in AE might expand to less recognized antibody-mediated conditions in the near future. We could recently demonstrate that asymptomatic mothers of a child requiring psychiatric in-patient diagnostics carried low-level pathogenic human NR1 antibodies more frequently than control mothers having a healthy child [36]. To better understand this possible connection, we developed a murine model of pregnancy-related materno-fetal antibody transfer. Here, human monoclonal NR1 antibodies diaplacentally transferred to the offspring, enriched in the fetal circulation and brain, caused neurotoxic effects during neonatal development, inducing brain network changes, and led to neuropathological disorders in the offspring persisting into adulthood [36]. Given the relatively high frequency of NR1 autoantibodies in the healthy human population, the findings indicate a novel disease principle with high clinical relevance for lifelong neuropsychiatric morbidity in the affected children [37]. Most importantly, these pathologies are potentially treatable with apheresis in asymptomatic mothers, but further studies are needed to better understand the frequency of autoantibodies, the susceptible window during pregnancy and the contribution of genetic and further risk factors. Further, this seems not to be limited to the NMDAR, as other maternal anti-neuronal autoantibodies may similarly cause neurodevelopmental disorders in the offspring, such as with antibodies against Caspr2 [38]. 

The use of therapeutic apheresis has been shown to be safe in pregnant women, both for mother and fetus. A recent Italian study evaluated the use of apheresis during pregnancy. Among 48 pregnant women receiving apheresis one had suspected autoimmune encephalitis. Adverse events occurred in 2.1% of all patients analysed, which is reported to be lower than the Italian average. Peripheral veins are preferred as a vascular access during pregnancy to avoid the risks associated with a central venous catheter [39].

### 3.6. Apheresis in Children with AE

Children with autoimmune disorders such as antibody-mediated AE can also be treated with therapeutic apheresis. PE was retrospectively evaluated in 22 children. All children had been treated with IVIG and/or steroids before PE. Each patient received a median number of six PE sessions. No PE-related mortality was observed and adverse events occurred in 2.2%, which is the expected average. Adverse events consisted of hypotension and urticaria. In total, three pediatric patients with antibody-mediated encephalitis were treated with PE, two patients improved and one patient showed partial recovery with persistent neurological deficits after three-year follow-up. One patient with paraneoplastic encephalitis did not benefit from PE and was lost to follow-up [40]. Another prospective observational study evaluated 535 children with acquired demyelinating syndrome or encephalitis treated with steroids, IVIG or PE. Here, pediatric patients with autoimmune encephalitis other than acute disseminated encephalomyelitis (ADEM) had the highest frequency of poor outcome. However, the individual treatment decisions were not specified [41].

### 3.7. Autoimmune Dementia and Treatment with Apheresis

Compared to ‘classic’ AE with subacute onset of neuropsychiatric as well as behavioral symptoms, antibody-associated dementias are a more slowly progressing group of diseases where decline in working and short-term memory as well as visuo-spatial deficits are the most prominent features. Detection of high-level autoantibodies in patients with dementia is rare, but autoimmune dementias represent a form of cognitive decline that is potentially treatable. A Mayo clinic study reported improvement of cognition in 64% of patients with suspected autoimmune dementia after immunomodulatory treatment [29]. An underlying autoimmune mechanism and response to immunotherapy is more likely when patients do not fulfill routine criteria for established neurodegenerative dementia forms, but rather present with subacute onset, psychiatric symptoms, fluctuating disease course, shorter delay to treatment, seropositivity for a specific autoantibody and inflammatory CSF [29].

In a retrospective study analyzing 286 CSF and serum samples of patients with different dementia forms, 16% of the serum samples had NMDAR IgA, IgM or IgG antibodies compared to 4.3% in a healthy control group [42]. Besides the spectrum of known and established pathogenic antibodies, there might be an even broader range of autoantibodies for which pathogenicity has not yet been confirmed. It is unclear from these studies whether anti-neuronal autoantibodies develop secondarily to neurodegeneration or whether they primarily contribute to and drive the disease. It is highly possible that neurodegeneration leads to the presentation of autoantigens from dying neurons with consecutive establishment of a specific autoimmune response. In this way, formed autoantibodies may contribute to synaptic dysfunction, further accelerate cognitive decline or contribute to clinical symptoms such as behavioral abnormalities commonly present in dementia patients.

Another recent target in dementia patients are autoantibodies against G protein-coupled receptors. In a small trial analyzing the effect of IA in patients with mild to moderate dementia and agonistic autoantibodies (agAAB) against α-adrenergic receptors, treatment with four cycles of IA not only caused disappearance of autoantibodies, but resulted in stabilization of the cognitive and mental condition during the follow-up period of 12–18 months [20]. Another study that is currently recruiting (ClinicalTrials.gov Identifier: NCT03132272) investigates the effects of IA in patients with Alzheimer’s disease positive for agAAB. The group aims to demonstrate discontinuation of the vascular remodeling and slowing of cognitive decline following IA treatment.

Apheresis not only has an acute effect on disease activity but can be used for longer lasting immunomodulation. In case of uncertainty of the immunological findings, first-line therapy including steroids or apheresis may serve as a diagnostic test to support the autoimmune etiology [43]. In our experience, however, short-term treatment (such as intravenous high-dose steroids for three to five days) cannot demonstrate clinical improvement in autoimmune dementia in most cases, thus requiring longer administration for four to six weeks (e.g., daily 0.5 mg/kg prednisone).

Based on the new developments in this field, the ongoing identification of dementia-associated autoantibodies and the concern about overlooking treatable etiologies, we now offer diagnostic antibody testing in serum and CSF to every patient with suspected dementia in our memory clinic at the department of neurology at Charité. In this way we increasingly identify patients with a working diagnosis of Alzheimer’s, frontotemporal dementia or atypical dementia who have new or established autoantibodies against neuronal and glial proteins, as exemplarily shown in the case vignette of an 81-year-old gentleman (Box 1).

Box 1 **Clinical case of autoimmune dementia.**

**Figure 2:**
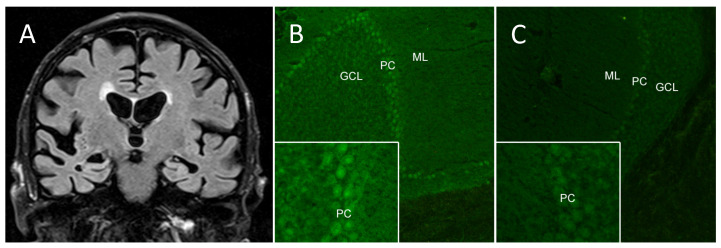
Autoantibodies against neurochondrin. (**A**) MRI T2/FLAIR (fluid-attenuated inversion recovery) of the 81-year-old dementia patient was largely unremarkable apart from few microangiopathic lesions in the left temporoparietal lobe. (**B**) Indirect immunofluorescence of murine cerebellum sections demonstrated CSF IgG antibodies against neurochondrin (GCL, granule cell layer; PC, Purkinje cells; ML, molecular layer). (**C**) Following immunotherapy with five sessions of IA, antibody titers in CSF were markedly reduced.

An 81-year-old dementia patient presented to our outpatient memory clinic at the department of Neurology at Charité—Universitätsmedizin Berlin with deficits in working and short term memory as well as difficulties in concentration. Symptoms began nine months prior to presentation with increasing loss of orientation for place and time, confusion and reported visual hallucinations. Brain MRI at symptom onset was unremarkable apart from microangiopathic lesions in the left temporoparietal lobe (Figure 2A). Basic CSF analysis showed markedly increased protein. Symptoms improved after several weeks without specific therapy, but anterograde memory deficit persisted. During the following months, two episodes with re-appearance of confusion occurred, but lasted only for days.

In our center, the patient showed a persisting dysexecutive syndrome with amnestic and visuo-constructive deficits and apraxia. No other neurological deficits were observed. Montreal cognitive assessment showed mild cognitive impairment with a score of 21 out of 30. A second CSF analysis revealed normal cell count, but still increased protein of 1288 mg/L (normal <450 mg/L). No infectious cause was found. Extensive search for anti-neuronal autoantibodies in serum and CSF including indirect immunofluorescence staining on rodent brain sections detected neurochondrin IgG antibodies in the CSF (Figure 2B).

The diagnosis of AE with oligosymptomatic memory deficits was established. A three-day course of intravenous methylprednisolone with 1 g/day led to improved gait, but no impact on cognition was observed. Because of the persisting memory deficits, two months later five sessions of IA were administered every other day and resulted in reduction of CSF neurochondrin antibody titers (Figure 2C). The patient reported vertigo during IA, but vital signs were unremarkable at all times. After IA, the patient described improved concentration; his wife reported better organization of daily life and his ability to care for himself again. In the Bristol Activities of Daily Living Scale the patient improved from 20 points to 9 points.

The patient’s autoantibodies targeted neurochondrin, a leucine-rich protein expressed not only in the brain, but also in bones and cartilage. As neurochondrin is located intracellularly, the pathogenicity of neurochondrin antibodies is unclear and they might only be a biomarker of autoimmunity including T-cell-mediated neurotoxicity [44]. Neurochondrin expression is highest in cerebellar Purkinje cells, brainstem, lateral parts of the central amygdala nuclei and the hippocampal pyramidal cells. Antibodies against neurochondrin bind robustly to the hippocampus, cerebellum and amygdala, while binding to the striatum, thalamus and cerebral cortex is less pronounced [45]. Patients described so far presented with rapidly progressing cerebellar ataxia, brainstem signs and neuropsychiatric symptoms.

Detection of antibodies against neurochondrin (Figure 2) in this patient led to immunotherapy with IA. The observed clinical improvement prompted B-cell depleting therapy with rituximab that led to long-term stabilization.

**Figure 3:**
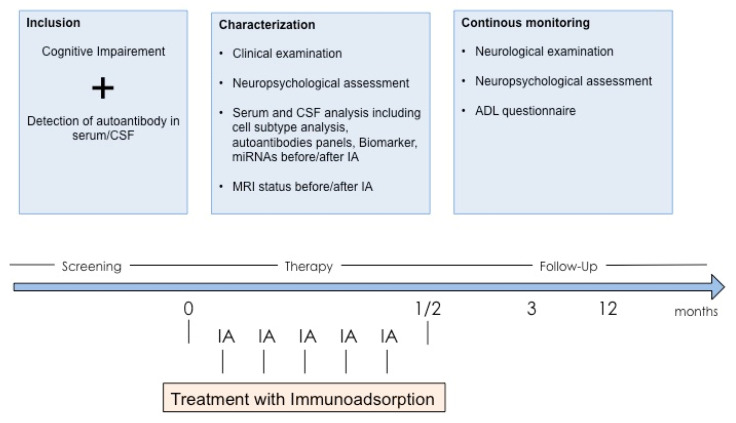
Immunoadsorption in autoantibody-positive patients with cognitive impairment—trial protocol. Patients with confirmed autoantibodies against central nervous system antigens and progressing cognitive impairment receive immunotherapy with five sessions of IA together with detailed neuropsychological, MRI and CSF biomarker assessment. Follow-up monitoring includes two visits after three and 12 months.

The patient participates in an ongoing clinical trial (DRKS00016017) analyzing the role of anti-neuronal and anti-glial surface antibodies in cognitive disorders and potential improvement following IA. The study aims to identify dementia patients who harbor autoantibodies against structures of the central nervous system using cell-based assays for detection of established autoantibodies as well as screening assays using indirect immunofluorescence on unfixed murine brain sections. Autoantibody-positive patients with cognitive decline can enroll in the study and receive therapeutic apheresis (Figure 3). Treatment includes five to six IA sessions over a 12-day course. Cognitive performance is evaluated prospectively and compared to historic controls. Patients further undergo structural und functional MRI before and after IA. CSF analysis evaluates the reduction of autoantibody levels over the course of IA and the potential utility of further biomarkers of neurodegeneration, such as micro-RNAs.

### 3.8. Closing Remarks and Outlook

Predictors for beneficial outcome after treatment with apheresis in patients with AE include start of the treatment early in the disease before substantial irreversible brain damage has occurred. Nevertheless, after longer periods from symptom onset to therapy initiation, apheresis can also result in symptom improvement. Immunotherapy with steroids or IVIG prior to apheresis does not seem to have an effect on the overall outcome. Especially in patients with severe disease courses apheresis is a major treatment option and should be initiated early, possibly together with other immune therapies.

For selected patient groups such as children and pregnant women as well as patients requiring ICU treatment, the safety and efficacy of apheresis could also be shown.

According to all studies reviewed, the ASFA guidelines and the recommendations given by the German Society of Neurology, no benefit of apheresis in patients with onco-neuronal antibodies could be shown. Here, results are inconsistent, with most patients showing no therapeutic effect after apheresis, or even further deterioration, but in single patients clinical improvement could sometimes be seen. A clear treatment response to apheresis, both, PE and IA, is established in patients with antibodies against surface antigens or synaptic antigens. In the articles screened for this review, there was no difference in outcome of patients treated with PE or IA. Therapeutic apheresis is a valuable option within the complex multimodal immune therapy of AE. The benefit of treating patients with antibody-mediated AE with apheresis by far outweighs the possible side effects that were not severe and were mainly associated with the central venous catheter. Furthermore treatment with apheresis may be complicated by the necessity of an ICU setting and poor patient cooperation.

Although predominant humoral autoimmunity seems to be rare in dementia patients and requires further study, the search for autoantibodies in these patients allows the detection of potentially treatable dementia forms and holds the potential to prevent further cognitive decline in selected patients. Thus, therapeutic apheresis is not only an important first-line therapy in patients with AE but may be increasingly considered in further patients who are positive for autoantibodies against neuronal structures. This already includes patients with cognitive decline but may in the future expand to novel clinical indications ranging from antibody-associated psychosis to autoantibody-positive pregnant women.

## Figures and Tables

**Table 1:** Most important antibodies and clinical syndromes.

Antigen	Clinical Presentation	Age/Gender	Tumor Type
Antibodies against neurotransmitter receptors [6]			
NMDAR [7]	Schizophreniform psychosis, perioral dyskinesia, epileptic seizures, coma, dystonia, hypoventilation	All ages, peak in childhood and youth, 75% women	Ovarian teratoma
GABAaR	Epileptic seizures, schizophreniform syndrome, refractory status epilepticus and epilepsia partialis continua	Younger adults; m > f (1.5:1)	Hodgkin lymphoma
GABAbR	LE with frequent epileptic seizures	Older adults f = m	50% lung cancer (SCLC)
AMPAR	LE, Epileptic seizures, memory deficits, psychosis	Older Adults f > m (2.3:1)	In 70% lung/breast cancer
mGluR5	LE, Ophelia syndrome (depression, agitation, hallucination, memory deficits, personality changes)	Young adults, m > f (1.5:1)	Hodgkin lymphoma
GlycinR	PERM (progressive encephalomyelitis with rigidity and myoclonus), SPS, cognitive deficits	Older adults f = m	Thymoma (<10%)
DPPX	LE with tremor, myoclonus, hallucinations, therapy refractory diarrhea	Older adults f < m (1:2.3)	Not known
Antibodies against ion channel subunits or cell adhesion molecules [8,9]			
LGI1	Facio-brachial dystonic seizures (FBDS), amnesia, psychosis, LE, hyponatremia	Adults > 40 years, m > f (2:1)	Rare
Caspr2	LE, neuro-myotonia, Morvan syndrome, can slowly progress over up to 1 year;similar to LGI1, but no hyponatremia	Elderly m > f (9:1)	Thymoma possible
IgLON5	REM- and non-REM sleep disorders, sleep apnea, stridor, dysarthria, dysphagia, dysautonomia, movement disorders, dementia	Older adults, f = m	Not known
Antibodies against intracellular (onconeural) antigens [10,11]			
Hu (ANNA-1)	Encephalomyelitis, brainstem encephalitis, LE, Denny-Brown syndrome	Large variability, depending on tumor type	>90%, SCLC
Ri (ANNA-2)	OMS, CS, encephalomyelitis		>90%, Ovary, breast cancer
Yo (PCA-1)	CS		>90%, Ovary cancer
Ma2	LE, CS, diencephalic/hypothalamic involvement		>90%, Testicular, lung cancer
CV2 (CRMP5)	Encephalomyelitis, LE, CS		>90%, SCLC, thymoma
Amphiphysin	SPS		>90%, Breast, SCLC
GAD	SPS, LE, ataxia	Middle aged, f > m (4:1)	Tumor association rare

LE: limbic encephalitis, SPS: Stiff-person syndrome, OMS: Opsoclonus-myoclonus syndrome, CS: cerebellar syndrome, SCLC: small cell lung cancer, PCD: paraneoplastic cerebellar degeneration.

**Table 2:** Overview of studies on therapeutic apheresis in autoimmune encephalitis (AE).

Author	Year	Journal	Study Type	AE Type	Sample Size	Procedure	Outcome Measurement	Results	Ref.
DeSena AD	2015	J Clin Aph	Retrospective	NMDAR	10	PE	Modified Rankin scale (mRS)	Steroids alone not as effective as steroids followed by PE	[17]
Ehrlich S	2012	Nervenarzt	Retrospective	Antibody-mediated, paraneoplastic	30	PE, IA	mRS	Improvement of mRS after PE or IA	[18]
Heine J	2016	J Neurol	Prospective	NMDAR, LGI1, Caspr2, GAD, mGluR5, Hu	21	PE, IA	mRS	Improvement of mRS in 60% of patients	[19]
Hempel P	2016	Ther Apher Dial	Prospective	agAAB	8	IA	Neuropsychological test	Stabilized cognitive performance after 4-day treatment	[20]
Köhler W	2014	Eur J Neurol	Retrospective	NMDAR, GABA, LGI1, GAD	13	IA	mRS	Improvement of mRS in 11/13 patients	[21]
Onugoren MD	2016	Neurol Neuroimmunol Neuroinflamm	Retrospective	LGI1, Caspr2, NMDAR, GAD	19	IA	mRS	Improvement of mRS in patients with LGI1, Caspr2, NMDAR, no improvement in patients with GAD	[22]
